# Influence of N-glycosylation on Expression and Function of Pseudorabies Virus Glycoprotein gB

**DOI:** 10.3390/pathogens10010061

**Published:** 2021-01-12

**Authors:** Melina Vallbracht, Barbara G. Klupp, Thomas C. Mettenleiter

**Affiliations:** Institute of Molecular Virology and Cell Biology, Friedrich-Loeffler-Institut, 17493 Greifswald-Insel Riems, Germany; Melina.Vallbracht@fli.de (M.V.); barbara.klupp@fli.de (B.G.K.)

**Keywords:** herpesvirus, pseudorabies virus, glycoprotein B, membrane fusion, N-linked glycosylation, virus entry

## Abstract

Envelope glycoprotein (g)B is conserved throughout the *Herpesviridae* and mediates fusion of the viral envelope with cellular membranes for infectious entry and spread. Like all viral envelope fusion proteins, gB is modified by asparagine (N)-linked glycosylation. Glycans can contribute to protein function, intracellular transport, trafficking, structure and immune evasion. gB of the alphaherpesvirus pseudorabies virus (PrV) contains six consensus sites for N-linked glycosylation, but their functional relevance is unknown. Here, we investigated the occupancy and functional relevance of N-glycosylation sites in PrV gB. To this end, all predicted N-glycosylation sites were inactivated either singly or in combination by the introduction of conservative mutations (N➔Q). The resulting proteins were tested for expression, fusion activity in cell–cell fusion assays and complementation of a gB-deficient PrV mutant. Our results indicate that all six sites are indeed modified. However, while glycosylation at most sites was dispensable for gB expression and fusogenicity, inactivation of N154 and N700 affected gB processing by furin cleavage and surface localization. Although all single mutants were functional in cell–cell fusion and viral entry, simultaneous inactivation of all six N-glycosylation sites severely impaired fusion activity and viral entry, suggesting a critical role of N-glycans for maintaining gB structure and function.

## 1. Introduction

Herpesviruses are large-enveloped double-stranded DNA viruses that include many important human and animal pathogens. Members of the *Alphaherpesvirinae* subfamily have the broadest host range, are neurotropic and can persist in the infected hosts for life [1]. In addition to the human herpes simplex viruses 1 and 2 (HSV-1, -2) and Varicella zoster virus (VZV), this subfamily contains many economically important veterinary pathogens such as the *Varicellovirus* Pseudorabies virus (PrV, *Suid alphaherpesvirus 1*). PrV is the causative agent of Aujeszky’s disease in swine and is capable of causing lethal disease in a variety of other mammalian species [2,3].

Infectious entry and spread of herpesviruses require the fusion of the viral envelope with the host cell membrane. Whereas many enveloped viruses encode a single but multifunctional glycoprotein (g) for host–cell receptor-binding and membrane fusion [4], herpesviruses utilize a multiprotein machinery to mediate these processes. The so-called “core-fusion machinery” which is conserved in all herpesviruses is formed by gB, the bona fide fusion protein, and the heterodimeric complex of the membrane-bound gH and anchorless gL (gH/gL) [5]. In addition to the core-fusion machinery, herpesviruses require the assistance of nonconserved subgroup-specific receptor-binding proteins such as gD of the *Alphaherpesvirinae* for entry [6,7,8].

Herpesvirus-mediated membrane fusion is a highly regulated process whose molecular details remain incompletely understood. The current model for alphaherpesvirus entry proposes that gD, gH/gL and gB drive membrane fusion in a cascade-like, pH-independent fashion [9,10,11]. Binding of gD to an appropriate host cell receptor, such as nectin-1 or herpesvirus entry mediator (HVEM), serves as the initial fusion trigger [12,13]. A conformational change in receptor-bound gD is proposed to allow its crosstalk with gH/gL, possibly by direct interaction between their ectodomains [14]. The gH/gL complex is believed to function as a fusion regulator and, upon triggering, is thought to activate gB, the sole fusion executor [5,9,11,15]. Although the exact molecular mechanism of gB fusion activation by gH/gL is unclear, direct interactions between the respective ectodomains and/or cytoplasmic domains have been reported to be essential for fusion activation [16,17]. Activated gB is proposed to facilitate fusion by refolding from a trimeric metastable high-energy pre-fusion conformation to a stable post-fusion state. During the fusogenic conformational change, gB exposes two internal fusion loops that interact with the target membrane [18]. The subsequent fold-back process into the energetically more favorable post-fusion conformation juxtaposes the viral and cellular membranes, ultimately leading to their fusion [5].

**Figure 1:**
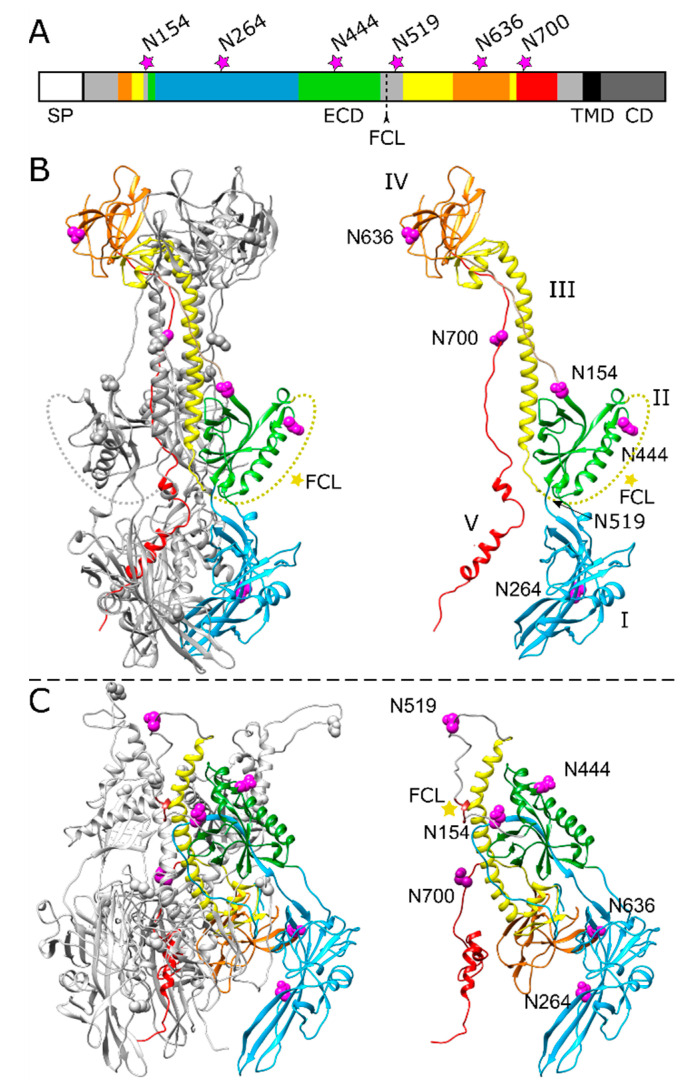
Position of potential N-linked glycosylation sites in the PrV gB ectodomain. (**A**) Schematic diagram of PrV gB with the signal peptide (SP), ectodomain (ECD), transmembrane domain (TMD) and a cytoplasmic domain (CD) indicated. The five domains (DI-V) forming the ectodomain are colored in blue (DI), green (DII), yellow (DIII), orange (DIV) and red (DV) according to the ribbon diagrams in (**B**,**C**), and regions not resolved in the post-fusion structure are depicted in grey. Position of the furin-cleavage site (FCL) and the potential asparagine (N)-linked glycosylation sites N154, N264, N444, N519, N636 and N700 are indicated. (**B**) Ribbon diagram of the PrV gB post-fusion trimer (PDB ID: 6ESC) [18], (left panel) and the monomer (right panel) is shown. Domains I–V of one protomer are highlighted in different colors, and the predicted glycosylation sites are indicated by purple spheres. N519 lies within a flexible region (dotted yellow line) that was not solved in the crystal structure and is marked by an arrow. The yellow star indicates the furin-cleavage site. (**C**) Model of pre-fusion PrV gB trimer (left panel) was generated using the protein structure homology-modeling server SWISS-MODEL [26] and the pre-fusion structure of HSV-1 gB as template [24]. Domains I–V of one protomer (right panel) are colored as in (**A**,**B**), and the predicted glycosylation sites are shown as purple spheres, and the furin-cleavage site is marked by a yellow star. The structure images were generated using UCSF Chimera (version 1.13.1) [27].

gB is the most highly conserved component of the herpesvirus fusion machinery and exhibits around 50% amino acid (aa) sequence identity within each subfamily [5]. High-resolution crystal structures of the stable post-fusion state have been determined for gB ectodomains of five different herpesviruses, including PrV [18,19], HSV-1 [20], and VZV [21], all revealing rod-shaped trimers which are composed of five domains (DI-V) per protomer (Figure 1). Based on these structural studies, gB was identified as a class III fusion protein [22]. In contrast to the well-characterized post-fusion conformation, structural information on the gB pre-fusion conformation is limited to HSV-1 [23,24] and the betaherpesvirus human cytomegalovirus (HCMV) gB [25].

The majority of viral envelope proteins, including herpesvirus gB, gH and gD, are modified by asparagine (N)-linked glycosylation, which can play crucial roles in correct folding, trafficking, function and immune evasion [28,29,30]. N-glycosylation starts in the endoplasmic reticulum (ER) with the en bloc attachment of oligosaccharides to the asparagine side chain in N-X-threonine (T) or -serine (S) sequons of the nascent polypeptide chain (X represents any amino acid except proline) [31]. Following the linkage of high mannose-type glycans to N of each sequon in the ER, glycan maturation takes place in the Golgi apparatus. Glycan processing involves trimming of glucose and mannose residues and subsequent addition of various terminal sugars resulting in different classes of glycans, including high mannose, hybrid and complex N-glycans [28,32]. Functional and structural studies on N-linked glycans from several viral fusion proteins, including influenza hemagglutinin [33,34], coronavirus spike protein [35,36,37,38], Ebola virus glycoprotein GP [39], envelope glycoprotein (Env) of human immunodeficiency virus-1 (HIV-1) [40,41], or envelope (E) protein of the flaviviruses Zika [42] and Dengue virus [43] have shown that these structures can play diverse functional and structural roles, e.g., in immune evasion by shielding from neutralizing antibodies, enhancement of infection, or stability and maturation of the respective fusion protein.

Recently, we have investigated the occupancy and functional relevance of N-glycosylation sites in PrV gH, highlighting a modulatory but non-essential role of N-linked glycans for gH function during membrane fusion [30]. In contrast to gH, there is only limited information on the role of N-linked glycans on gB. In fact, the only gB homolog tested for the role of all predicted N-linked glycans is HSV-2 gB [29]. Therefore, in the present study, we set out to expand our analyses to the N-linked glycans in PrV gB. PrV gB contains six potential N-glycosylation sites, all located in the ectodomain. Occupation of three predicted sites by N-glycans (N264 in DI, N444 in DII, N636 in DIV) was confirmed by crystal structure analysis of the PrV gB post-fusion ectodomain, but their functional relevance is unknown.

To investigate the significance of N-glycosylation on PrV gB, we systematically inactivated all six potential sites by the introduction of a conservative mutation in the individual sequons in the plasmid cloned gB gene (UL27), resulting in the replacement of asparagine (N) by glutamine (Q). Resulting gB mutants were tested for correct expression and glycosylation as well as fusogenicity in a virus-free transfection-based cell–cell fusion assay [44]. In addition, the ability of the gB variants to function in virus entry was investigated by transcomplementation of a gB-deficient PrV mutant.

## 2. Results

### 2.1. Effect of N-Glycosylation Site Mutations on gB Processing and Expression

The amino acid sequence of PrV gB strain Kaplan (Ka) contains six potential N-linked glycosylation sites matching the consensus motif N-X-T/S, where X represents any amino acid (aa) except proline [31]. All predicted N-glycosylation sites map to the gB ectodomain (Figure 1). The three sites at aa positions N154, N264, N444 reside within the N-terminal subunit of furin-cleaved gB, while sites N519, N636 and N700 are located within the C-terminal subunit (Figure 1A–C). Crystal structure analysis of the PrV gB post-fusion ectodomain expressed in insect cells revealed occupation of three N-glycosylation sites, namely N264 in domain I (DI), N444 in DII and N636 in DIV [18]. Based on the recently published pre-fusion structure of HSV-1 gB, a model of PrV pre-fusion gB was generated using the protein structure homology-modeling server SWISS-MODEL [26], and the N-glycosylation sites were mapped into the structure (Figure 1C). For further analysis, the N codon of each sequon was replaced by a glutamine codon (N➔Q) via site-directed mutagenesis of the gB expression plasmid. The resulting gB expression plasmids were used for in vitro expression, transient-transfection-based fusion assays and trans-complementation studies.

**Figure 2:**
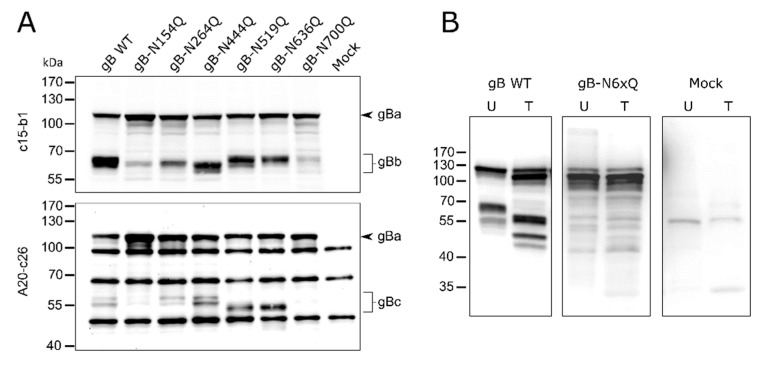
Expression and glycosylation analysis of PrV gB N-glycosylation mutants. (**A**) Lysates of RK13 cells transfected with expression plasmids for the indicated gB variants were separated by SDS–PAGE under reducing conditions. Lysates of cells transfected with empty vector pcDNA-3 served as a negative control (Mock). Blots were either probed with the monoclonal antibody (mAb) c15-b1, directed against the N-terminal subunit (gBb) of furin-cleaved gB (upper panel), or with mAb A20-c26 (lower panel) recognizing the C-terminal gB subunit (gBc). Signals of uncleaved gB (gBa) or furin-cleaved gB subunits (gBb and gBc) are labeled by arrowheads and braces. The molecular masses of marker proteins are indicated. Representative blots from three independent experiments are shown. (**B**) Analysis of N-linked carbohydrates in gB. Lysates of RK13 cells expressing PrV wild-type (WT) gB or gB-N6xQ were either left untreated (U) or treated (T) with PNGase F. Samples were separated by SDS–PAGE under reducing conditions, and blots were probed with a monospecific rabbit antiserum against PrV. Three independent experiments were performed, and one representative blot is shown.

The occupancy of the individual N-glycosylation sites and processing of the different gB mutants was investigated by Western blot analyses of cell lysates from rabbit kidney (RK13) cells transfected with expression plasmids for gB wild-type (WT) and the different N-glycosylation site mutants. Fully glycosylated PrV gB is an approx. 120-kDa polypeptide that undergoes proteolytic processing by cellular furin in the Golgi apparatus [45,46]. The protease recognizes the basic sequence ^501^RRARR^505^, located in a flexible loop that connects gB DII and DIII (Figure 1). Cleavage after R505 generates two subunits, gBb (~69-kDa) and gBc (~58-kDa), which remain linked by disulfide bonds. To visualize PrV gB in Western blot analyses two monoclonal antibodies (mAb) (c15-b1 and A20-c26) were used that detect either the N-terminal (gBb, c15-b1) or the C-terminal subunit (gBc, A20-c26), respectively (Figure 2) [18,47].

As expected, two bands were observed for PrV WT gB, corresponding to the uncleaved gB precursor (gBa) and the N-terminal subunit (gBb) when mAb c15-b1 was used (Figure 2A, upper panel), or the C-terminal subunit (gBc) when blots were probed with A20-c26 (Figure 2A, lower panel). The mutants revealed WT-like behavior in processing by furin with the exception of gB-N154Q and gB-N700Q (Figure 2A). These variants showed only low levels of the furin-cleaved subunits and a more abundant signal for the uncleaved gB precursor indicating that N154Q and N700Q affected furin-cleavage (Figure 2). Inactivation of the N-glycosylation sites at N264 and N444 resulted in a shift of the N-terminal subunit (gBb) to a lower molecular mass, indicating that both sites are occupied. Differences in the molecular weight between gBb of the two single mutants gB-N264Q and gB-N444Q suggested differences in the added N-glycans (Figure 2A, upper panel). For N519Q or N636Q, which are located in the C-terminal subunit, higher electrophoretic mobility of the respective gBc could be observed when compared to WT gB (Figure 2A, lower panel). These results indicate that gB positions N264, N444, N519 and N636 are occupied by N-glycans. Mutation of the N-glycosylation site N700Q and, to a lesser extent, N154Q had an impact on gB processing by cellular furin.

In addition, a gB variant was generated in which all six N-glycosylation sites were inactivated simultaneously (gB-N6xQ). Cell lysates of gB-N6xQ expressing RK13 cells were treated with peptide-N-glycosidase F (PNGase F), which removes all N-linked carbohydrates. Similar to the single mutant gB-N700Q, only the uncleaved gB precursor could be observed for gB-N6xQ. However, unlike PrV gB WT, gB-N6xQ was not sensitive to PNGase F treatment and revealed a similar apparent molecular mass as gBa of PNGase F gB WT (Figure 2B). Overall, these results show that all predicted N-glycosylation sites on PrV WT gB are used and that they are inactivated in gB-N6xQ.

**Figure 3:**
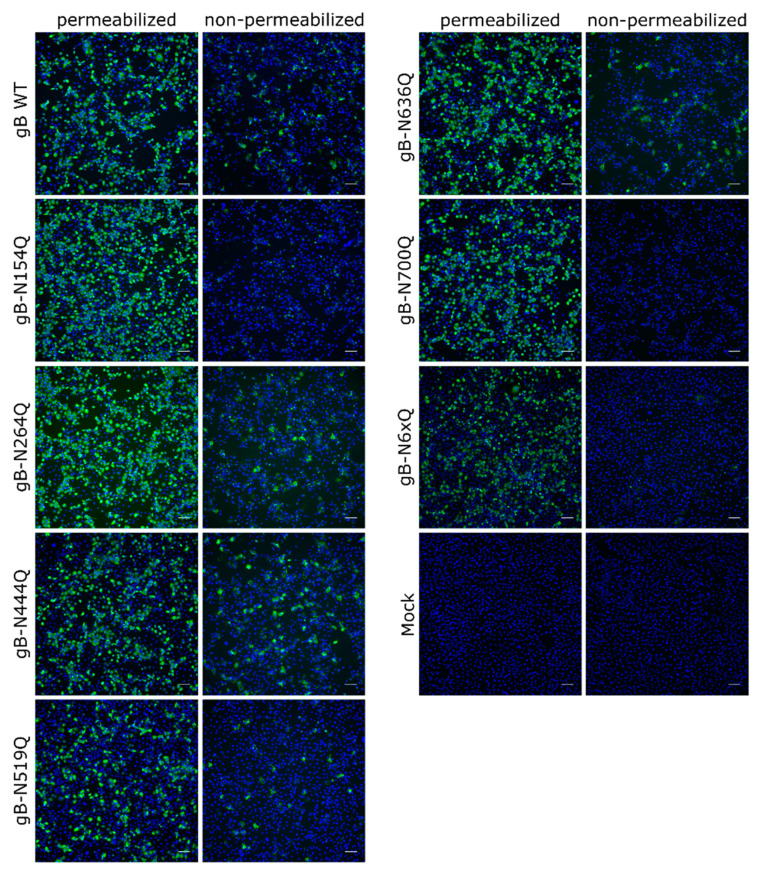
Subcellular and surface expression of the PrV gB N-glycosylation mutants. RK13 cells were transfected with expression plasmids for wild-type (WT) gB or the indicated gB mutants. One day after transfection, cells were fixed and either permeabilized or not. gB was detected using a gB-specific rabbit antiserum and Alexa Fluor 488-conjugated secondary antibodies. Nuclei were stained with Hoechst. Total and cell surface expression of gB was analyzed by fluorescence microscopy (Nikon Eclipse Ti-S). Scale bars: 100 µm. Representative images from three independent experiments are shown.

To investigate the expression and subcellular localization of the different mutants, indirect immunofluorescence analyses of transfected RK13 cells using the polyclonal anti-gB serum [48] without or after permeabilization were performed (Figure 3). Low levels of surface localization could be observed for PrV WT gB, which is in line with previous results [18]. Fluorescence-activated cell sorting (FACS) analysis revealed that only around 4% of PrV WT gB is presented at the plasma membrane (data not shown), complicating robust quantitation. Surface localization of gB-N264Q, -N444Q, -N519Q and -N636Q was similar to WT gB. However, no staining could be detected without permeabilization for mutants gB-N154Q, gB-N700Q or gB-N6xQ, indicating that gB N-glycosylation at N154 and N700 is not only important for processing by cellular furin but also for gB surface localization.

### 2.2. In Vitro Fusion Activity of gB N-Glycosylation Mutants

**Figure 4:**
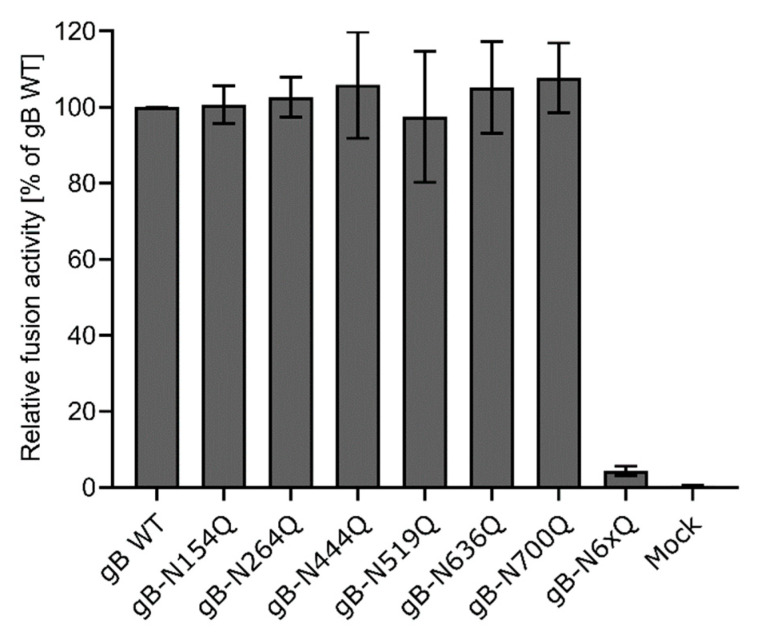
Cell–cell fusion activity of gB N-glycosylation site mutants. RK13 cells were cotransfected with 200 ng of the expression plasmids for enhanced green fluorescent protein (EGFP), PrV gH, gL, gD, gB wild-type (WT) or the indicated gB glycosylation site mutant. Cells transfected with EGFP and empty vector pcDNA-3 served as a mock control. 24 h post-transfection, syncytia formation was assessed. The number of syncytia in 10 fields of view was multiplied by the corresponding mean syncytia area to obtain the total fusion activity. Fusion activities were normalized to activities obtained with the four wild-type glycoproteins. Mean relative values from four independent experiments and corresponding standard deviations are shown.

To investigate the role of the six N-glycosylation sites for gB function during membrane fusion, the different mutants were tested in a transient transfection based cell–cell fusion assay [44]. This assay exploits the ability of the herpesvirus entry glycoproteins gB, gH/gL and gD to mediate cell–cell fusion in the absence of other viral components, thereby allowing for more direct analysis of gB function during fusion than in the viral context (Figure 4).

Fusion activities of the different gB mutants in combination with gH/gL and gD were determined 24 h after cotransfection of RK13 cells with the respective expression plasmids and enhanced green fluorescent protein (EGFP), which was used as a marker to visualize syncytia formation. The area and number of syncytia within 10 fields of view (5.5 mm^2^ each) were determined, and the mean syncytia area was multiplied by the number of syncytia to obtain the absolute fusion activity. Fusion activity of the four wild-type proteins served as positive control and was set as 100%, while empty vector pcDNA-3 was substituted for the gB expression plasmid and used as a negative control. Although inactivation of N154 or N700 affected furin-cleavage and surface localization of gB as evident from Western blot analyses and immunofluorescence analyses (Figure 2A and Figure 3), all mutants revealed wild-type like fusion activities (Figure 4). In contrast, simultaneous inactivation of all N-glycosylation sites severely affected gB fusogenicity. Fusion activity of gB-N6xQ was almost reduced to background levels (Figure 4), suggesting that N-glycans may be important for the overall gB structure and/or stability of the protein.

### 2.3. Impact of gB N-Glycosylation Site Mutations on Virus Entry

**Figure 5:**
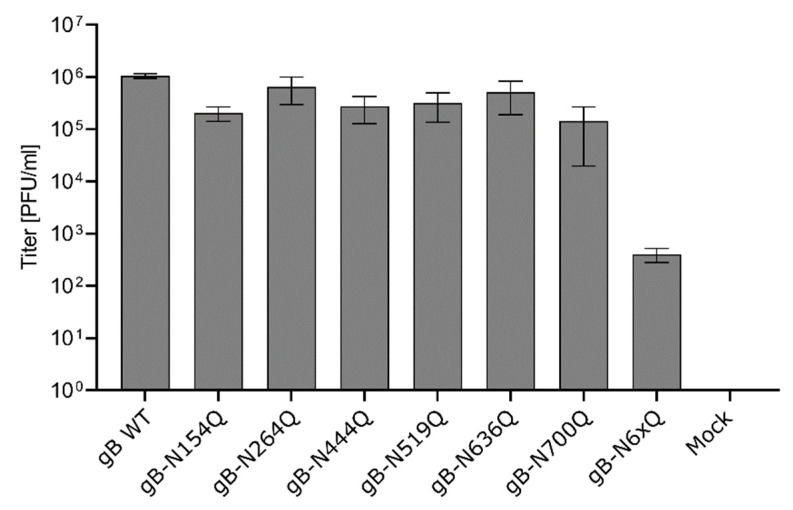
Virus entry mediated by the gB N-glycosylation site mutants. RK13 cells were transfected with expression plasmids encoding WT or mutant gB. Cells transfected with an empty vector served as a negative control (Mock). 24 h later, the cells were infected with PrV-ΔgB at an MOI of 3. Progeny virus titers were determined on gB-expressing RK13 cells and are given in plaque-forming units (PFU)/ml. Shown are the mean values from four independent experiments and corresponding standard deviations.

The ability of the gB mutants to function in virus entry was investigated by complementation of a PrV mutant lacking the gB gene UL27 (PrV-ΔgB) [47]. To this end, RK13 cells were transfected with expression plasmids for either WT gB, the different gB mutants or with the empty vector pcDNA-3, which served as a negative control. One day post-transfection, cells were infected with trans-complemented PrV-ΔgB at an MOI of 3. After 24 h, cells and supernatants were harvested, and progeny virus titers were determined on RK13 cells stably expressing PrV gB WT (RK13-gB) [47]. In line with the results from cell–cell fusion assays, all gB mutants with a single inactivated N-glycosylation site were able to efficiently complement the gB-deficient PrV mutant to titers comparable to WT gB (Figure 5). In contrast, complementation by gB-N6xQ resulted in severely reduced titers of only approximately 5 × 10^2^ PFU/ml. However, although very low, titers of gB-N6xQ complemented PrV-ΔgB were still above background levels (Figure 5), indicating a residual level of gB fusion activity even in the absence of all N-linked glycans.

## 3. Discussion

Host-cell-derived N-linked glycans on viral envelope proteins can influence protein folding and stability, transport, viral entry and spread, and immune evasion [49]. Recently, we investigated the functional relevance of N-linked glycosylation in PrV gH [30]. In the present study, we extended our analyses to N-linked glycans on PrV gB. To this end, the six predicted N-glycosylation sites in PrV gB (N154, N264, N444, N519, N636 and N700) (Figure 1) were systematically inactivated by conservative mutation (N➔Q). The resulting single mutants and a gB mutant with inactivation of all six predicted sites were tested for proper expression, processing, and function in cell–cell fusion and viral entry.

**Figure 6:**
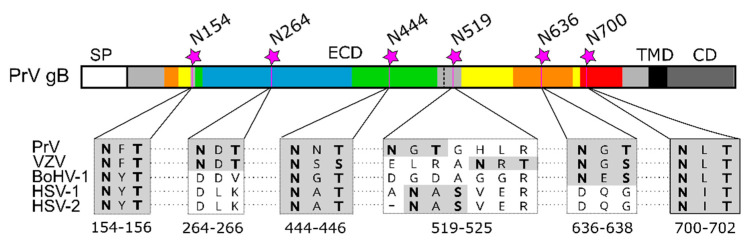
N-glycosylation sites in alphaherpesvirus gB. Schematic representation of the PrV gB open reading frame colored and labeled as in Figure 1A. N-linked glycosylation sites N154, N264, N444, N519, N636 and N700 in PrV gB are indicated, and corresponding sequence alignment of the N-X-S/T sequons of gB of pseudorabies virus (PrV; EM64049.1), varicella-zoster virus (VZV; AH010537.2), bovine alphaherpesvirus 1 (BoHV-1; KU198480), human alphaherpesvirus 1 (HSV-1, strain F; ADD59998.1), human alphaherpesvirus 2 (HSV-2, strain 333; ABU45421.1) are shown below. The N-X-S/T sequons are shown in boldface on gray background. The sequence alignment was generated using ClustalW (Geneious Prime 2019.2.3).

Comparative sequence analyses reveal that three out of six sites in PrV gB, namely N154, N444 and N700, are conserved among alphaherpesvirus gBs, N519 is not present in BoHV-1, N636 is conserved in members of the Varicellovirus genus, and N264 has only a counterpart in VZV (Figure 6).

N154 is the most N-terminally located N-linked glycosylation site in PrV gB and maps to a flexible region connecting domains II and III (Figure 1 and Figure 6). Western blot analyses showed that inactivation of this site affected the processing of gB by cellular furin, as evident by low levels of the two furin-cleaved subunits gBb and gBc and a more abundant signal for the uncleaved gB for this mutant (Figure 2A). Early studies revealed that gB cleavage occurs in the Golgi apparatus after gB oligomerization in the ER [45]. Herpesvirus gB is believed to undergo extensive conformational changes for fusion and is reported to transit from a (predicted) trimeric pre- to a trimeric post-fusion form [5]. Only recently, the pre-fusion structure of HSV-1 gB was reported [24]. Here, the HSV-1 pre-fusion gB structure was used as a template to generate a model for pre-fusion PrV gB (Figure 1C). In contrast to the location of aa N154 in the post-fusion conformation (Figure 1B), in the PrV gB pre-fusion model, N154 is positioned in close proximity to the furin-cleavage site (Figure 1C). Thus, it is conceivable that inactivation of this site resulting in the absence of the carbohydrate moiety at N154 may have an influence on the surrounding protein structure, which affects the accessibility for or recognition by furin and results in the observed defect in the cleavage. Despite impaired cleavage and cell surface localization (Figure 2A), gB-N154Q was still able to mediate efficient in vitro cell–cell fusion to levels comparable to WT gB (Figure 4). Moreover, this mutant was able to rescue the entry defect of a gB-deficient PrV mutant (PrV-ΔgB) reaching titers similar to WT gB complemented PrV-ΔgB (Figure 5). Although proteolytic cleavage is a prerequisite for fusion activity of several viral fusion proteins such as influenza hemagglutinin, paramyxovirus F or retrovirus Env, protease cleavage is not required for fusion activation of PrV gB [18,50], which is also true for other class III fusion proteins [51,52]. Thus, our results parallel previous findings that proteolytic cleavage is not required for PrV gB function [18]. Moreover, the data confirm earlier studies showing that in vitro fusion activity and surface expression of gB do not correlate [47,53]. Interestingly, mutation of N133 in HSV-2, which corresponds to PrV gB N154, was similarly shown to affect protein maturation and intracellular trafficking, suggesting a conserved function of this site [29].

Similar to site N154, N700, which is the most C-terminally located gB N-glycosylation site and maps to DV, was found to be important for proper processing by furin and cell surface localization (Figure 2A and Figure 3). Interestingly, site N700 is also conserved in alphaherpesvirus gBs and corresponds to N668 in HSV-2 gB (Figure 6). As observed for N700 in this study, inactivation of glycosylation at N668 impaired processing of HSV-2 gB [29]. However, in contrast to PrV gB in which sites N154 and N700 are apparently dispensable for in vitro fusion (Figure 4) and viral entry (Figure 5), inactivation of the glycosylation sites at N133 or N668 significantly inhibited in vitro fusion and viral entry mediated by HSV-2 gB [29]. These findings highlight similarities but also intriguing differences between gB of the two viruses.

Differences in the requirements for gB fusion activity exist [5] and early swapping experiments have shown that PrV gB could complement the lethal defect of a gB-negative HSV-1 mutant, but not vice versa [54]. Moreover, whereas HSV-1 gB induced only very low levels of in vitro cell–cell fusion when coexpressed with PrV gH/gL and gD, fusion activity observed for PrV gB with the HSV-1 glycoproteins was significantly higher [55]. These experiments indicate that fusion mediated by PrV gB is more robust and may require fewer specific interactions than gB of HSV, further suggesting that PrV gB may be more tolerant of local structural changes.

Two other N-linked glycosylation sites in HSV-2 gB, N390 and N483, have been found to be important for in vitro cell–cell fusion activity and viral entry [29]. However, the site at N390 is conserved only in the simplex viruses, and PrV gB N519, corresponding to N483 in HSV-2 gB (Figure 6), was found to be dispensable for in vitro fusion activity and viral entry of PrV gB (Figure 4 and Figure 5), indicating different roles of this site in gB of the two viruses. When the study by Luo et al. was conducted [29], the pre-fusion structure of HSV-1 gB was not available. However, in the HSV-1 pre-fusion structure and in the pre-fusion PrV gB model, aa N483 (HSV-2 gB) and N519 (PrV gB) are exposed on the very top of the molecule (Figure 1C). Due to this position, it is tempting to speculate that a glycan attached to this site could play a role in immune evasion.

Western blot analyses indicated that sites N264, N444, N519 and N636 are all occupied by N-glycans since the respective N-terminal (gBb), or C-terminal (gBc) subunits migrated faster compared to the respective WT gB fragments (Figure 2A). Although these sites are apparently all modified by N-glycans in PrV gB, their presence is not required for proper gB processing, surface localization (Figure 3), function during cell–cell fusion (Figure 4), or viral entry (Figure 5). Nevertheless, they may perform different biological functions in vivo. Interestingly, the three single mutants gB-N154Q, gB-N264Q and gB-N444Q exhibited differences in their respective molecular weights when compared to each other (Figure 2A, upper panel). In particular, gB-N444Q migrated faster than gB-N154Q and gB-N264Q. It is well established that glycoproteins can be heterogeneous in their content of complex N-glycans, whereby the N-glycan diversity may be caused by the aa sequence and/or the local structure influencing substrate availability for Golgi glycosidases or glycosyltransferases [56]. Thus, the observed differences in molecular weight may be due to the heterogeneous composition of carbohydrates attached to N444, N154 and N264 [57].

N636 is located in gB DIV and is part of an NXS/T sequon that is conserved among members of the varicelloviruses VZV and BoHV-1 but not in the simplex viruses HSV-1 and 2 (Figure 6). N620 of VZV gB, which corresponds to PrV N636, lies within a conserved beta-strand in DIV, which was recently reported to be part of an epitope recognized by a neutralizing human mAb (93k) directed against VZV gB [21]. Although inactivation of N636 had no effect on PrV gB function during cell–cell fusion or viral entry, the relevance of this N-glycosylation site in vivo remains to be determined. In this context, it is well documented that N-glycans of viral surface proteins, e.g., of HIV-1 Env, can interfere with the host antibody response by serving as a “shield” to cover essential epitopes, thereby protecting the virus from antibody-mediated neutralization [58]. At present, we cannot exclude that N-glycans on PrV gB could serve to obscure epitopes, which could be targeted by neutralizing antibodies.

In addition to the single mutants, a gB variant was generated in which all N-glycosylation sites were inactivated simultaneously. Glycosylation analysis revealed that this gB variant is not sensitive to PNGase F digestion (Figure 2B), and gBa bands of PNGase digested and non-treated gB-N6xQ migrated comparable to PNGase F-treated WT gB, indicating that all sites were indeed inactivated in gB-N6xQ (Figure 2B). Western blot (Figure 2B) and indirect immunofluorescence analyses (Figure 3) revealed that gB-N6xQ is neither processed by furin nor detectable on the surface of transfected RK13 cells. Although all single mutants were able to promote WT-like in vitro cell–cell fusion and viral entry, gB-N6xQ was incapable of mediating efficient cell–cell fusion, and fusion activity was reduced to almost background levels (Figure 4). In good correlation with these results, gB-N6xQ complemented gB-deficient PrV to only very low titers of 5 × 10^2^ PFU/mL (Figure 5). For other components of the herpesvirus fusion machinery, such as HSV-1 gD, it was shown that the absence of N-glycans influences the overall protein structure. However, these structural changes did not affect gD function during infection in cell culture [59,60,61]. With respect to this, it is conceivable that the individual mutations in PrV gB led to minor structural changes that have no major impact on gB structure and subsequently gB functionality. In gB-N6xQ, however, the structural changes may have an additive adverse effect on gB structure, leading to impaired processing and loss of function.

In summary, our results show that all six predicted N-glycosylation sites in PrV gB are modified in vitro by N-glycans. We demonstrate that the individual N-glycosylation sites play a modulatory but dispensable role for gB function, whereas simultaneous inactivation of all sites severely affects gB function in fusion, highlighting an important role of these glycans for correct folding, structure and/or stability of gB. Although mutation of most of the sites had no notable effect on gB processing and function during in vitro fusion and entry, which is largely congruent to our results on the role of N-glycans on PrV gH [30], we found that N-glycosylation of PrV gB at the highly conserved positions N154 and N700 is important for proper processing of gB by cellular furin. Taken together, this study expands our knowledge on the role of N-linked glycans on the herpesvirus fusion protein gB. Which role gB N-glycosylation plays in vivo and whether removal of certain sites can affect virus-specific antibody responses remains to be determined.

## 4. Materials and Methods

### 4.1. Cells and Viruses

Rabbit kidney (RK13) and RK13-gB cells [47] were grown in Dulbecco′s modified Eagle′s minimum essential medium (MEM) supplemented with 10% fetal calf serum at 37 °C. Viruses used in this study were derived from PrV strain Kaplan (PrV-WT) [62]. The PrV mutant lacking the gB gene has been described previously [47].

### 4.2. Expression Plasmids and Generation of PrV gB N-Glycosylation Mutants

Generation of expression plasmids for PrV-WT gB, gD, gH, and gL has been described previously [63]. The expression plasmid encoding PrV gB WT was used for site-directed mutagenesis (QuikChange II XL kit, Agilent, CA Santa Clara, USA). PrV gB residues are numbered according to GenBank accession number AEM6404.1 [64]. The oligonucleotide primers used for gB mutagenesis leading to inactivation of the potential N-glycosylation sites are listed in Table 1. Correct mutagenesis was verified by sequencing using the standard primers T7 and SP6 as well as the gB-specific primers 130 and 134 listed in Table 1, the BigDye Terminator v1.1 cycle sequencing kit, and a 3130 genetic analyzer (Applied Biosystems, CA Foster City, USA).

### 4.3. Western Blot Analyses

RK13 cells were harvested 24 h after transfection with 600 ng of the expression plasmids encoding wild-type gB or the different N-glycosylation mutants using lipofectamine 2000 (Thermo Fisher Scientific, Darmstadt, Germany). Cells were lysed, and protein samples were separated by discontinuous sodium dodecyl sulfate-polyacrylamide gel electrophoresis (SDS–PAGE) and transferred to nitrocellulose membranes. Membranes were incubated with monoclonal PrV gB antibodies (c15-b1 or A20-c26 at 1:10 dilution) [18,47] and peroxidase-conjugated secondary antibody (Dianova, Hamburg, Germany). Clarity Western ECL substrate (Bio-Rad Laboratories, Feldkirchen, Germany) and a VersaDoc 4000 MP imager (Bio-Rad Laboratories, Feldkirchen, Germany) were used for detection.

### 4.4. PNGase F Digestion for Glycosylation Analysis

Lysates of RK13 cells transfected with expression plasmids for WT gB or gB-N6xQ were treated with 500 U of peptide-N-glycosidase F (PNGase) for 2 h at 37 °C under buffer conditions according to manufacturer′s instructions to remove all N-linked glycans. After digestion, samples were separated by SDS–PAGE as described above.

### 4.5. In Vitro Cell–Cell Fusion Assays

Fusion activity of the gB N-glycosylation site mutants was determined using a transient transfection-based cell–cell fusion assay [44]. Briefly, approximately 1.8 × 10^5^ RK13 cells per well were seeded into 24-well cell culture plates and transfected with 200 ng each of the expression plasmids for EGFP (pEGFP-N1; Clontech), PrV gH, gL, gD and WT gB or the different N-glycosylation site gB mutants in 100 μL of Opti-MEM using 1 µL of lipofectamine 2000 (Thermo Fisher Scientific, Düsseldorf, Germany). Twenty-four hours post-transfection, cells were fixed with 3% paraformaldehyde, and syncytium formation was assessed using an Eclipse Ti-S fluorescence microscope and NIS-Elements imaging software (Nikon, Düsseldorf, Germany). Total fusion activity was determined by multiplication of the number of syncytia with three or more nuclei by the mean syncytia area within 10 fields of view (5.5 mm^2^ each). Mean values and standard deviations from four independent assays were determined.

### 4.6. Trans-Complementation Assay

The ability of the different gB mutants to function in virus entry was determined by trans-complementation of PrV-ΔgB [47] as described previously [18]. Briefly, approximately 1.8 × 10^5^ RK13 cells per well were seeded into 24-well cell culture plates. On the following day, cells were transfected with 400 ng of the expression plasmid for WT or mutant gB using 1 µL lipofectamine 2000. Twenty-four hours post-transfection, the cells were infected with phenotypically gB-complemented PrV-ΔgB at a multiplicity of infection (MOI) of 3 on ice and consecutively incubated 1 h on ice and at 37 °C for 1 h. Subsequently, the inoculum was removed, and the non-penetrated virus was inactivated by low-pH treatment [65]. Cells were washed with PBS, and 1 mL fresh prewarmed medium was added to the monolayer. After twenty-four hours at 37 °C, the cells were harvested together with the supernatants and lysed by freeze-thawing (−80 °C and 37 °C). Progeny virus titers were determined on RK13-gB cells. The experiment was repeated four times, and mean values and corresponding standard deviations were calculated.

### 4.7. Comparative Indirect Immunofluorescence Analysis

To visualize total and surface localization of the gB mutants, comparative indirect immunofluorescence analyses of transfected and permeabilized or non-permeabilized RK13 cells were done. RK13 cell monolayers in 24 well cell culture dishes were transfected with 400 ng of the corresponding gB expression plasmids as described above (lipofectamine 2000; Thermo Fisher Scientific, Darmstadt, Germany). Twenty-four hours post-transfection, cells were fixed with 3% PFA in PBS for 20 min. Optionally, cells were permeabilized in PBS containing 0.1% Triton X-100 for 10 min at room temperature. Subsequently, all cells were washed with PBS and blocked with 0.25% skimmed milk in PBS before they were incubated with the rabbit antiserum specific for PrV gB at a dilution of 1:1000 in PBS [48]. After 1 h at room temperature, bound antibody was detected with Alexa 488- or 633-conjugated goat anti-rabbit antibodies (Thermo Fisher Scientific, Darmstadt, Germany) at a dilution of 1:1000 in PBS. Nuclei were stained with Hoechst (33,342 staining dye solution; Abcam, Berlin, Germany) at a dilution of 1:20,000 in PBS for 10 min at room temperature. Representative images were taken with a Leica DMi8 fluorescence microscope (Leica Microsystems, Wetzlar, Germany).

## Figures and Tables

**Table 1:** Oligonucleotides used for mutagenesis and sequence analyses.

Primer Name	Sequence (5′➔3′)
gB Ka N154Q F	CTCGCAGGGGCGC**CAG**TTCACGGAGGGG
gB Ka N264Q F	CGGCTGGCACACCACC**CAG**GACACCTACACC
gB Ka N444Q F	CGGCGGCGCTAC**CAG**AACACGCACGTGCTGG
gB Ka N519Q F	CGCCGGCCGTC**CAG**GGCACGGGGCACC
gB Ka N636Q F	CACCTTCGAGCAC**CAG**GGCACGGGCGTG
gB Ka N700Q F	CGCGGGTGACCCTG**CAG**CTGACGCTGCTGG
130	CGTGCCCGTCCCCGTGCAGGAGATC
134	CCATCTACCGGCGGCGCTACAACAG

Only forward strand mutagenesis primers (F) are listed. The corresponding reverse strand primers are exactly complementary. PrV gB residues are numbered according to GenBank accession number EM64049.1 [64]. Nonmatching nucleotides are shown in boldface.

## Data Availability

Data are contained within the article.
